# Fabrication of Novel Bentonite-Anthracite@Zetag (BT-An@Zetag) Composite for the Removal of Arsenic (V) from an Aqueous Solution

**DOI:** 10.3390/molecules27217635

**Published:** 2022-11-07

**Authors:** Mohamed R. El-Aassar, Ahmed K. Alezbaway, Ibrahim O. Althobaiti, Mohamed Y. El-Sayed, Hend S. Abu Salem, Hassan M. A. Hassan, Rawan F. Alolaimi, Emam F. El Agammy, Mohamed S. Mohy-Eldin, Fathy M. Mohamed

**Affiliations:** 1 Chemistry Department, College of Science, Jouf University, Sakaka 72341, Saudi Arabia; 2 Geology Department, Faculty of Science, Helwan University, Ain Helwan 11795, Egypt; 3 Chemistry Department, College of Science and Arts, Jouf University, Al-Nasfah 77217, Saudi Arabia; 4 Geology Department, Faculty of Science, Cairo University, Giza 12613, Egypt; 5 Physics Department, College of Science, Jouf University, Sakaka 72341, Saudi Arabia; 6 Polymer Materials Research Department, Advanced Technology and New Material Institute, City of Scientific Research and Technological Applications (SRTA City), New Borg El-Arab City 21934, Egypt; 7 Hydrogeology and Environment Department, Faculty of Earth Sciences, Beni-Suef University, Beni-Suef 62521, Egypt

**Keywords:** bentonite, anthracite, zetag, As(V), composites, arsenic, water treatment, water pollutant

## Abstract

The arsenic (As) pollution of water has been eliminated via intensive scientific efforts, with the purpose of giving safe drinking water to millions of people across the world. In this study, the adsorption of As(V) from a synthetic aqueous solution was verified using a Bentonite-Anthracite@Zetag (BT-An@Zetag) composite. The SEM, FT-IR, XRD, DSC, TGA, and SBET techniques were used to characterize the (BT-An@Zetag) composite. The adsorption of As(V) was explored using batch adsorption under varied operating scenarios. Five kinetic modelswere used to investigate kinetic data, whereas three isotherms had been used to fit empirical equilibrium data. According to the findings, the adsorption mechanism of As(V) was best described by the Freundlich isotherm with a maximum monolayer coverage of 38.6 mg/g showing pseudo-second-order mode. The estimated enthalpy (H°) indicates that the adsorption process is both chemical and endothermic.The calculated free energy (G°) indicates that the reaction is nonspontaneous. After four sequential adsorption cycles, the produced BT-An@Zetag composite demonstrated good reusability and a greater adsorption affinity for As(V) ions. Overall, the BT-An@Zetag composite is suited for removing arsenic from wastewater using adsorption as a cost-effective and efficient technique.

## 1. Introduction

As a result of anthropogenic activities and natural processes, arsenic (As) has been discharged into surface water and groundwater, causing severe health concerns to humans and other living organisms. In many parts of the world, arsenic exposure has been associated with significant health risks such as kidney, bladder, lung, and skin cancers, as well as other skin diseases [1]. Toxic arsenic is consumed by 35–50 × 10^6^ people in Bangladesh and West Bengal, more than ten million in Vietnam, and more than twenty million in China, according to current estimates [2,3]. Arsenic is often included in insecticides, glass, desiccants, alloys, pigments, electronic components, and pharmaceuticals, as well as wood preservatives in recent years, all contributing to arsenic poisoning [4,5]. Arsenic is mostly abundant in the aquatic environment as arsenate and arsenite species, with arsenate (As(V)) being the most prevalent in oxidizing conditions [6]. The presence of As(V) in natural water is a consequence of the leaching of arsenic-rich rocks and sediments [7,8]. Because arsenic is a known carcinogen as well as a toxic element, its presence in drinking water, even at trace levels, causes a health risk. The permitted limit of arsenic in potable water, according to the World Health Organization (WHO) and the United States Environmental Protection Agency, is 10 μg/L [9,10,11].

Several studies were made to effectively remove arsenic from water, e.g., by activated biochar [12,13], natural iron-enriched samples [14], activated red mud [15], and rare-earth based materials [16]. The adsorption method is the most promising method due to its easy handling, sludge-free operation, and efficient use in various systems [11]. Activated alumina is an effective adsorbent used for arsenic removal from polluted water [17,18,19,20]. Because of the following advantages, adsorption has gotten a lot of attention in comparison to other conventional procedures such as coagulation/precipitation, oxidation, ion exchange, filtration, membrane/reverse osmosis, and biological treatment. Generally, (i) it does not require a large volume or additional chemicals, (ii) it is easier to set up as a POE/POU (point of entry/point of use) arsenic resorption system [21], and (iii) it produces no hazardous byproducts [22,23] and is potentially more cost effective [14,24]. Many researchers used volarized anthracite in wastewater treatment [25].Anthracite impregnated with carbon nanotube (MW/CNT) [26]. For example, acrylonitrile derivatives [27], poly (Styrene-co-Acrylonitrile) copolymer [28], activated silica [29], composite of olive seed residue, anthracite, chitosan [25], inorganic coagulant of aluminum and iron impregnated with activated silica [30,31], and PFTE derivatives [32]. Other scientists conducted arsenic removal via different materialssuch as Sulphide Precipitation [33],Iron-Coated Cork Granulates [34,35], Iron-Coated Seaweeds [36], and Porous HematiteIron-Based Adsorbent [37,38].

The steps implemented in this study include: (1) the impregnation of bentonite with anthracite using Zetag binder, (2) the characterization of the obtained composite BT-An@Zetag, and (3) assessing the influence of experimental factors (pH, sorbent doze, As equilibrium concentration, mixing speed, and time) on the sorption of As(V) on BT-An@Zetag in a batch system (4) fitting the experimental results to the different kinetic and isotherm models to better understand the adsorption mechanism of As(V) [39,40,41]. Thus, the aim of this paper is to synthesize, for the first time, a novel BT-An@Zetag composite for As(V) removal from the aqueous solution and evaluate its adsorption behavior based on the isotherm, kinetics, and thermodynamics’ characteristics.

## 2. Results and Discussions

### 2.1. The BT-An@Zetag Composite Characterization

#### 2.1.1. XRD Analysis of the BT-An@Zetag Composite

**Figure 1:**
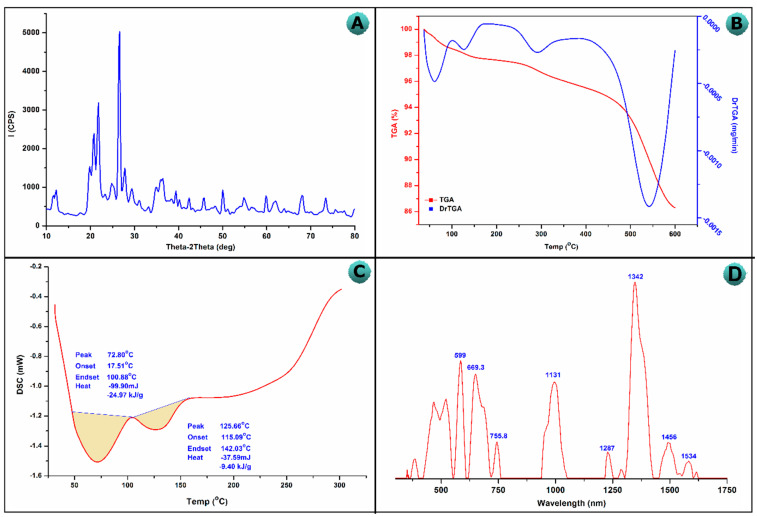
(**A**) XRD pattern, (**B**) TGA, (**C**) DSC, and (**D**) Raman spectra of the BT-An@Zetag composite.

The BT-An@Zetag composite X–ray data showed crystalline nature at 22° and 25° (Figure 1A). Furthermore, the SiO_2_ reflection was represented by the sharp diffraction peaks at 26° [42,43]. The crystalline structure of BT-An@Zetag composite was reflected by strong peaks at 24°, 29°, 42°and 45°. The crystalline polymorphs of silica cristobalite and tridymite, which were detected at 20.94, 36.64, 50.17, 60.11, 68.23, and 77.31°, correspond with the SiO_2_ peaks of native BT and BT-An@Zetag composite [44,45]. A strong peak at 26.5° and a weak peak at 43.9° suggested the existence of an amorphous graphite structure in the BT and BT-An@Zetag composites, and is attributed to the random lattice structure of anthracite layers [45].

#### 2.1.2. Thermogravimetric Analysis/Differential Scanning Calorimetry Analysis (TGA)

The TGA analysis of the BT-An@Zetag composite was shown in Figure 1B. The degradation of the BT-An@Zetag composite is multi-staged, with no stable intermediate product. The release of volatile gases such as water vapor, SO_2_, CO, CO_2_, and Zetag could account for weight losses of 1.5 and 3% at 50 and 300 °C, respectively. The TGA curve of the BT-An@Zetag composite shows three primary decomposition steps: the first is due to the loss of volatile gases or adsorbed water from Zetag, while the second is due to Zetag degradation and dehydration, as well as de-polymerization of the Zetag chain [46,47]. Conversely, in the DSC thermograms of the BT-An@Zetag composite, the volatile gases were lost at around 50–150 °C, according to the graph, and the BT-An@Zetag composite attained a steady state after this temperature (Figure 1C). Due to their high content, volatile elements, notably volatile gases, were lost around 50 and 150 °C in the BT-An@Zetag composite. Furthermore, the shape of the thermograms denotes an endothermic reaction.

#### 2.1.3. Raman Spectroscopy

Figure 1D shows the Raman spectrum of the BT-An@Zetag composite. The two conspicuous peaks G and D were attributed to the spectrum of (An), with the G band having a larger amplitude at 1585 cm^−1^, indicating the emergence of the graphite structure [48,49,50]. The addition of anthracite and Zetag may be responsible for the increased intensity of the BT-An@Zetag composite. This stage corresponds to C=C stretching in the aromatic ring’s hexagonal sheet. The Raman disorder shift is assigned to the Raman scattering mode’s dual resonance at 1250–1450 cm^−1^, while the Dband represents a lattice structure disorder as well as cavities in aromatic rings [51]. The disorder in the BT-An@Zetag composite graphite layer can be detected, which is in exact agreement with another recently published graphite material investigation [52,53]. These comments go hand in hand with the XRD results for the BT-An@Zetag composite [54,55].

#### 2.1.4. Surface Morphology and Elemental Analysis

**Figure 2:**
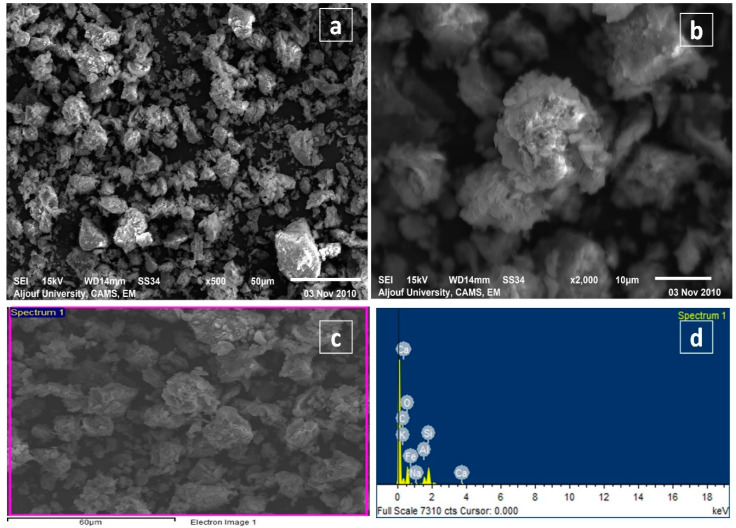
Scanning electron microscopy with the energy-dispersive X-ray spectroscopy (SEM-EDX) analysis of the BT-An@Zetag composite (**a**–**d**).

The elemental analysis was performed with a SEM-EDX micrograph in BT-An@Zetag, as shown in Figure 2a,b, respectively. Surface morphology was used to identify the inhomogeneous topography of minerals confined in a carbon matrix. The presence of C, O, Na, Mg, Al, Si, Ca, and Fe (C: 25.15, O: 54.47, Na: 0.74, Mg: 0.48, Al: 4.57, Si: 11.39, Ca: 0.75, S: 0.44, and Fe: 1.64%) for BT-An@Zetag was observed. Figure 2a,b shows the BT-An@Zetag SEM diagrams incorporating (BT-An) into the Zetag polymer; On the other hand, Figure 2a,b shows the production of the BT-An@Zetag composite with bright and opaque characteristics. SiO_2_ and Al_2_O_3_ were found as big particles with a diameter of 10 μm in BT-An@Zetag composites. The inclusion of anthracite and Zetag resulted in an increase in oxygen content and a decrease in carbon content in the BT-An@Zetag composite as a result of the binding with anthracite and Zetag. The increase in BT-An@Zetag composite holes leads to an increase in surface area (103.5 m^2^/g) after mixing with anthracite and Zetag at temperatures of 70 and 90 °C during the manufacturing and treatment of BT-An@Zetag composites. Volatile gases such as H2, CO, and CO_2_ evaporate at these temperatures, creating these gaps [42].

#### 2.1.5. N2-Adsorption Analysis

**Figure 3:**
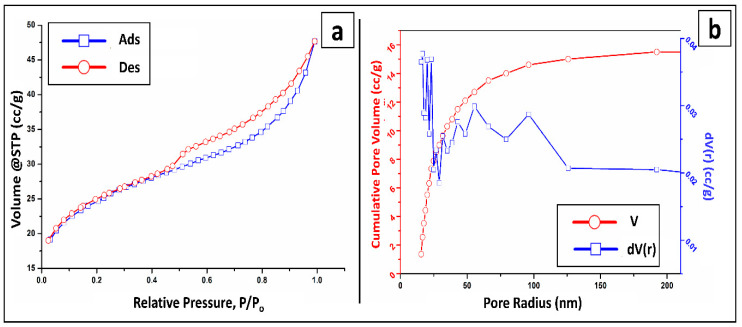
(**a**) N_2_adsorption/desorption isotherms curves of the BT-An@Zetag composite obtained from the nitrogen gas, and (**b**) the BJH pore size distribution of the BT-An@Zetag composite.

Figure 3a,b shows N2 adsorption/desorption isotherms produced at 77 K and BJH pore size distributions of BT-An@Zetag composite. The adsorption–desorption isotherms of Type I curves according to the IUPAC classification are shown in the BT-An@Zetag composite based on BET analysis, which is indicative of the presence of a narrow mesopore micro-porosity structure and a wider structure of micropores. The BT-An@Zetag composite surface area grew dramatically from 10.937 m^2^/g to 81.935 m^2^/g, implying a high micropore concentration in the composite BT-An@Zetag [56], with micropores gradually increasing from 0.00028 to 0.0151 (cm^3^/g). Following that, low-density volatile gases evacuated during the preparation and curing of the BT-An@Zetag composite, implying a tidy formation and resulting in a smaller hole between the layers. As a result of the pore curing, the specific surface area (SSA) and very microporous structure of the composite BT-An@Zetag will be dramatically developed, as well as the SSA. In addition, Figure 3b shows the BJH-measured pore size distributions of the BT-An@Zetag composite. The composite BT-An@Zetag has a tiny hole diameter distribution of 1.5–5 nm, with big pores ranging from 5 nm to over 50 nm. The results showed that curing created more pores; mesoporous materials may prevail in porous structures, therefore, curing resulted in an increase in the composite BT-An@Zetag’s total surface area, which is qualitatively compatible with the change in the surface area of the BET. The results demonstrate that due to a high temperature and increased degree of carbonization, the degree of the microcrystalline BT structure shifts greatly and enhance the degree of graphitization, potentially resulting in an excellent sorptive material [57]. The average pore size, total pore volume, and surface area obtained from the BT-An@Zetag composite are 13.87, 0.0333 cm^3^/g, and 81.935 m^2^/g, respectively, (Table 1).

### 2.2. Controls on As(V) Adsorption

#### 2.2.1. Effect of pH

**Figure 4:**
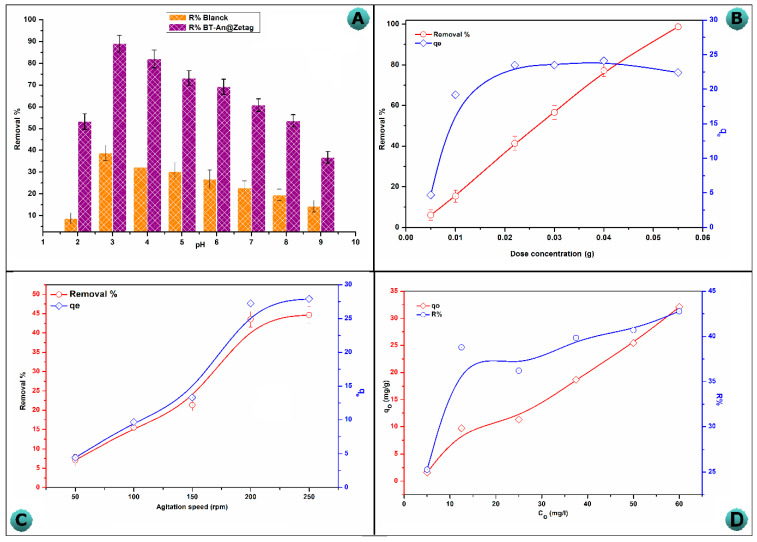
(**A**) The effect of solution pH on As(V) adsorption by BT-An@Zetag composites (Concentration: 50 mg/L, adsorbent weight: 20 mg, contact time: 120 min, and agitation speed: 200 rpm at 25 °C), and effect of (**B**) adsorbent doze, (**C**) agitation speed, and (**D**) initial concentration on As(V) adsorption by the BT-An@Zetag composite.

In general, the solution pH represents an important parameter influencing heavy metal adsorption because it affects the adsorbent’s surface charge as well as the prevailing species of the metal ions. The effect of altering the solution pH (3–9) on the adsorption of As(V) ions using BT and the BT-An@Zetag composite is shown in Figure 4A. At a pH of 3, As(V) had the highest adsorption capability onto BT (12.25 mg/g) and the BT-An@Zetag composite (27.8 mg/g). It is worth mentioning that under identical working conditions, As(V) had a higher adsorption capacity on the BT-An@Zetag composite than BT. However, increasing the pH from 4 to 7 reduced the adsorption capacity to 19 mg/g due to the partial deprotonation of the BT-An@Zetag composite’s functional groups, followed by a decrease in the interaction between the adsorbent’s oxygen/amine containing-functional groups and As(V). Furthermore, elevating the pH of the solution from 8 to 10 resulted in the competition for active sites on the adsorbent surface between functional groups (i.e., OH^−^) and AsO_4_^−3^ anions (the only stable oxidative form at this pH). H_2_AsO_4_^−4^ and HAsO_4_^−4^ compounds are native in all arsenate complexes [58].

Depending on the nature of the charge, these species will attract or repel on the adsorbent surface. The H_2_AsO_4_^−4^ and HAsO_4_^−4^ species adhere to the positive surface of the BT-An@Zetag composite, leading the negative charge on the adsorbent to grow while the positive charge diminished. For the similarity of charges, the surface of BT-An@Zetag should change to negative and repel the H_2_AsO_4_^−4^ and HAsO_4_^−4^ species, leading to a decrease in As(V) adsorption. Other researchers [59,60] noticed the same tendency in the influence of pH on arsenic eradication. At pH levels up to 7, the As(V) removal by the BT-An@Zetag composite was maintained at a high adsorption capacity. A chemical interaction, in addition to electrostatic contact, is a fair assumption.

#### 2.2.2. Effect of Adsorbent Dose

Figure 4B depicts the effect of BT-An@Zetag dosage on As(V) adsorption. Because there were more adsorptive sites for the ions to adsorb as the adsorbent dose increased from 5 to 55 mg, the amount of As(III) and As(V) adsorbed by BT-An@Zetag was still over 13 mg/g at 10 µg/L and over 40 mg/g at 50 µg/L [60]. However, increasing the BT-An@Zetag masses up to 55 mg had no impact on the As(V) adsorption efficiency (98.7%) due to the equilibrium between the adsorbate and the adsorbent under specific operating conditions, as well as the solid-concentrationeffect [61]. When the dose of BT-An@Zetag was increased from 5 to 55 mg, the arsenic ion adsorption capacity increased dramatically from 4.696 to 22.425 mg/g. The increase of the adsorbent mass to roughly 22 mg relatively maintains the adsorption capacity (23.49 mg/g). On the other hand, once the BT-An@Zetag dose is increased from 5 to 55 mg, the adsorption capacity decreases to 22.425 mg/g.

#### 2.2.3. The Agitation Speed Effect

The effect of increasing the speed of agitation (50–250 rpm) on the As(V) adsorption behavior in a 25 mL arsenic solution at PH = 3 and 25 °C is shown in Figure 4C. With an increase in agitation speed, the adsorption capacity and adsorption efficiency of arsenate ions rise from 4.44 to 27.91 mg/g and from 7.1 to 44.66 percent, respectively, as a result of an enhanced solute molecule dispersion and an increased exposed adsorbent surface area to the arsenate ions. When the speed is 250 rpm, however, the adsorption performance approaches constant values. As a result, the plateau refers to the removal of all accessible As(V) from the solution.

#### 2.2.4. The Initial Concentration Effects

In Figure 4D, the adsorption capacity and removal efficacy were plotted against the initial As(V) concentration. The adsorption performance improved, and it achieved its highest value (32.08 mg/g) with removal percentages of 42.8%when the As(V) concentration increased. These findings could be explained by the availability of more energetic spots at lower As(V) ion/adsorbent ratios, as well as the huge ratio of the adsorbent’s accessible surface area to the number of As(V) ions moles. The active sites, on the other hand, were rapidly occupiedby increasing the metal ion/adsorbent ratio, thus, decreasing the adsorption efficiency as the adsorption began at lower energy sites. The As(V) removal percentage diminishes from 42.8 to 25.3 percent as the initial As(V) concentration rises from 5 to 60 mg/L.

#### 2.2.5. Contact Time Effect

**Figure 5:**
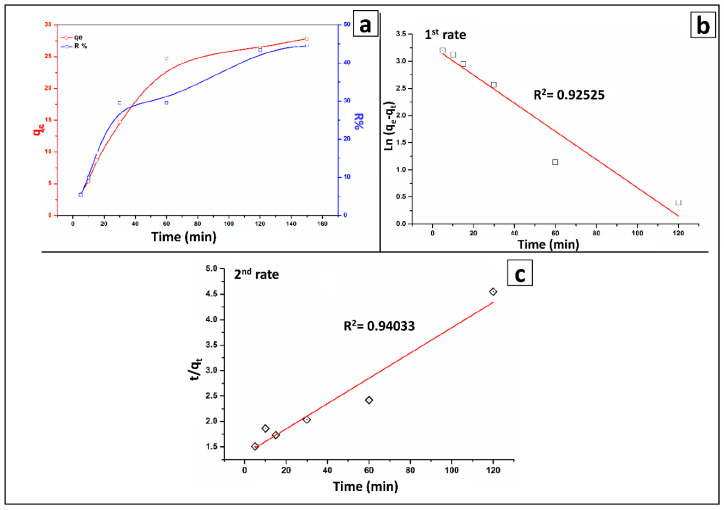
(**a**) Effect of time, (**b**) pseudo-first-order, (**c**) pseudo-second-order models for the adsorption of As(V) by the BT-An@Zetag composite.

Figure 5a shows the adsorption as a function of residence time (5–150 min) at a metal ion concentration of 25 mg/L, BT-An@Zetag dosage of 20 mg, and a pH of 3. The sorptive capacity and the percentage of arsenic adsorption obviously improved as the contact time was increased from 5 to 150 min, with the maximum adsorption capacity reaching 26.361 mg/g. The composition of the sorbent and the available surface sites had a significant impact on the time it took to reach equilibrium (which was120 min for BT-An@Zetag). Firstly, there may be more vacant active sites available for adsorption, however, as adsorption time passed [62], the repulsive forces between adsorbate molecules and the solid phase increased, making residual free surface sites difficult to occupy and the adsorption affinity decrease. The equilibrium times for 11.175 mg/L and 44.703 mg/L are 0.5 and 3 h, respectively.

### 2.3. Kinetics and Mechanism of the Adsorption Process

The estimated parameters of the preliminary kinetic models are summarized in Table 2. In comparison to the pseudo-second-order model, the q_cal_ value produced from the pseudo-second-order model (Figure 5b) was close to the value of q_exp_ (Figure 5a). Thus, the pseudo-second-order model best fits the kinetic sorption mode, highlighting that the adsorption of As(V) onto the BT-An@Zetag composite is advantageous. Furthermore, it is assumed that the rate of metal ion uptake is dependent on the number of active sites on the sorbent surface. The rapid adsorption rate (h) reveals that a significant number of arsenic ions accessed the sorbent surface in a short amount of time.

The Elovich model was used to assess the likelihood that different forces are relevantto the adsorption of As(V) from the system [39]. In this model, the greater constant value is related to the extension of surface coverage, indicating that the BT-An@Zetag adsorbent has a larger surface area (Table 2). The lower value of the constant, which is related to the chemisorption rate, suggests that more than one mechanism was involved in the uptake of arsenate.

**Figure 6:**
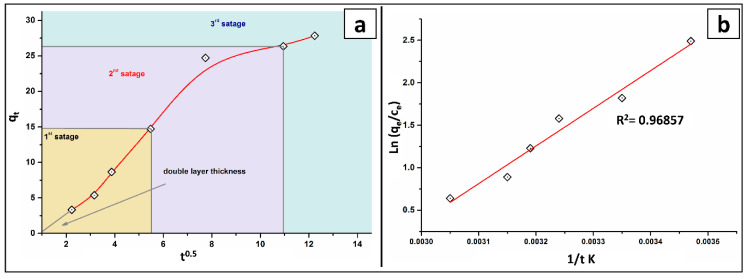
(**a**) Intraparticle-diffusion models and (**b**) the Van’t Hoff plot on the adsorption process of As(V) by BT-An@Zetag composite.

The intraparticle diffusion was employed to investigate the adsorption process of As(V). The many stages of the intraparticle-diffusion model for As adsorption are clearly depicted in Figure 6a. Additionally, the adsorption achievement was not constant over the entire time range, indicating that the adsorption process is governed by several phenomena. The multilinear plot did not pass through the origin, implying that As(V) adsorption’s process is complex, with both intra-particle diffusion (i.e., boundary-layer diffusion) and surface adsorption contributing to the rate limiting step.

The minor value of Kp1 (the slope of the first steeper stage) than the anticipated value of Kp2 (the slope of the second linear section) supports this finding, as do other authors [25]. Additionally, as demonstrated in Table 2 and Figure 6a, the intercept values are proportional to the depth of the border layer.

Additionally, the distinction between film and particle diffusion mechanisms is clearly shown by Boyd linear plot (Figure 6b). If the straight line passes through the origin, the adsorption rate is determined by particle diffusion; if not, it is regulated by film diffusion. Film diffusion is clearly revealed in the Boyd plots as the regulating mechanism. Other researchers [29] have observed similar investigations.

### 2.4. Thermodynamic Study

**Figure 7:**
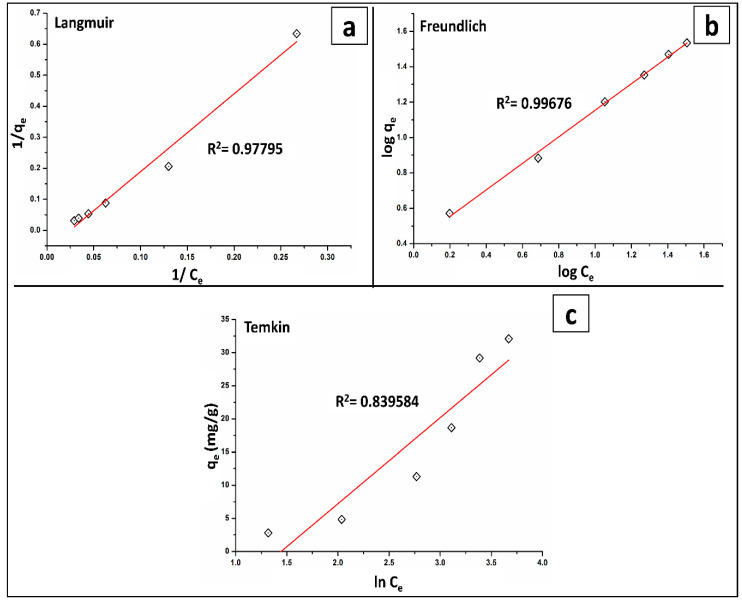
RAdsorption isotherm models of (**a**) Langmuir, (**b**) Freundlich, and (**c**) Temkin of As(V) by the BT-An@Zetag composite.

The mechanism of As(V) adsorption onto the constructed BT-An@Zetag composite was studied at temperatures ranging from 288 to 318 K, as illustrated in Figure 6b. The adsorption capacity decreased from 28.315 to 18.875 mg/g, suggesting that more energy was necessary to defeat the activation-barrier between the solid/liquid interfaces and allow more AsO_4_^−1^ ions to penetrate the BT-An@Zetag adsorbent. The decrease in adsorption ability could be attributed to a decrease in the potential of attraction between the adsorbate and the adsorbent, reflecting an endothermic mechanism. For the spontaneity of the adsorption phase, the thermodynamic parameters studied from the slope and intercept of the plot of lnK_D_ vs. 1/T shown in Figure 7b can be evaluated; Table 3. The positive enthalpy value (36.864 kJ/mol) revealed that chemisorption is As(V) adsorption, which is endothermic and advantageous at low temperatures. This result confirmed that the fundamental response between As(V) and BT-An@Zetag composites is complex and dominated by electrostatic interactions [25,39], (i.e., concurrent physical and chemical reactions). Furthermore, the negative entropy value (−107.53 J/mol K) during the adsorption of As(V) ions onto the BT-An@Zetag composite in an aqueous solution shows unpredictability at the liquid/solid mediator [41]. The positive value of G ranging from 67.934 to 71.058 kJ/mol) at the temperature range studied, on the other hand, confirms the likelihood of a non-spontaneous adsorption response. 

### 2.5. Isotherm’s Modeling

Table 4 lists the isotherm parameters that have derived from visualizing the three isotherm models shown in Figure 7a–c. The Langmuir isotherm is shown in Figure 7a. The Freundlich isotherm (Figure 7b) best described the absorption of As(V) onto BT-An@Zetag surface (R^2^ = 0.997), implying a monolayer adsorption with heterogeneous adsorption energy. Furthermore, the low value of K_L_ and high value of q_max_ (38.65 mg/g) indicate that the fabricated BT-An@Zetag composite has a considerable sensitivity for As(V). Additionally, the computed R_L_ value was less than 1, whereas the 1/n value calculated from the slope of the Freundlich model (Figure 7b) was greater than 1, indicating that As(V) adsorption was advantageous. Furthermore, the larger positive value of BT obtained from the Temkin plot indicated a greater sorbent/sorbate interaction (Figure 7c). In this investigation, the maximum monolayer uptake capacity of As(V) was compared to that of other adsorbents in the literature, with the findings reported in Table 5.

### 2.6. Evaluation of Adsorbent Reusability

**Figure 8:**
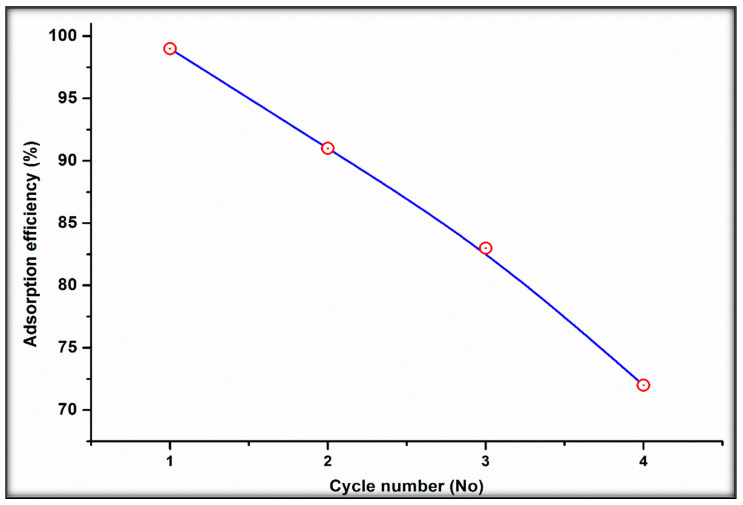
Reusability of BT-An@Zetag adsorbent composite.

The ability of sorbent materials to be rejuvenated and reprocessed multiple times is a critical economic aspect because it determines the cost of production. The immediate attraction between the metal ions and the adsorbent surface was recovered by regenerating the arsenic-adsorbed composite in this study. After four consecutive cycles, the BT-An@Zetag composite adsorbent retained a high uptake affinity, as shown in Figure 8, with As(V) removal (percent) still above 70 percent. The results show that the synthesized composite is a good As(V) adsorbent that is also recyclable.

### 2.7. Adsorption Mechanism

**Figure 9:**
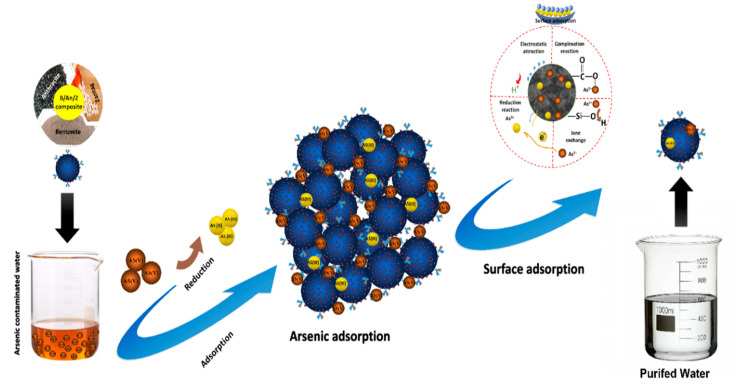
Mechanism of the As(V) adsorption by the BT-An@Zetag adsorbent composite.

The adsorption mechanism of As(V) on the BT-An@Zetag composite was shown in Figure 9. The electrostatic forces between the negatively charged As(V) species and the multi-functional groups of the composite, and the surface complexation with carboxyl and hydroxyl groups were discovered, and thus, the uptake mechanism of As(V) was reduced by the adjacent electron donor groups. During the adsorption process, the As(V) was converted to less harmful As (III) ions by the interaction between the negatively charged carboxyl groups of the BT-An@Zetag and the arsenic ions. Furthermore, by an ion exchange mechanism, the As(V) metal ions can be adsorbed by the Si-O group [63].

Electrostatic attraction was observed between positively charged adsorbent surfaces and negatively charged arsenate species, indicating that electrostatic forces may have played a role in the adsorption process. Metal ions and surface groups interact to absorb BT-An@Zetag particles and metal ions, according to the theory of surface chemistry.

Due to the exterior surface of the BT-high An@Zetag’s protonation at (pH 2.0–4.0), there are many attraction forces between (arsenate) species and functionalized groups of the material. One such force is electrostatic attraction, where the arsenate species and the protonated Si-OH^+^ of the BT-An@Zetag adsorbent showed the highest uptake percentages.

## 3. Materials and Methods

### 3.1. Materials

Natural Bentonite (BT) from the Egyptian bentonite clay quarries at the Egyptian Western Desert is used. Anthracite was supplied by Egypt’s Matrouh Water and Wastewater Company (An), which was characterized by El-Aassar et al. [25]. Polyelectrolyte Zetag used in this study is the commercial name and was obtained from dental waste, while sodium arsenate (Na_3_AsO_3_.7H_2_O) was obtained from Sigma-Aldrich, USA, at a purity of 99.5 percent. The stock solution (1000 mg/L) of As(V) was prepared by dissolving a specified weight of Na_3_AsO_3_.7H_2_O in deionized water. Sodium hydroxide (NaOH), and Hydrochloric acid (HCl) (37%) of analytical grade were obtained from El-Nasr Company, Egypt. All compounds used in this investigation were used exactly as they were supplied.

### 3.2. Preparation of Bentonite/Anthracite/Zetag (BT-An@Zetag) Composite

After just being dried at 90 °C for 48 h, 5 g of bentonite (BT) and 1 g of anthracite were carefully crushed and sieved below 100 microns. The obtained composite material was then softly crushed and sieved below 63 microns and classed as BT-An composite after being mixed with 0.5 g of Zetag, which was dissolved in 5 mL of distilled water and dried at 90 °C for 48 h.

### 3.3. Adsorbent Characterization

BT-An@Zetag composite samples were analyzed using an X-ray diffractometer (Shimadzu XRD-7000, Tokyo, Japan with an X-ray wavelength Cu detector). Scanning electron microscopy was used to analyze the morphological topographies of BT-An@Zetag composites (SEM, JEOL JSM-6610 LV, Tokyo, Japan). Thermal gravimetric analysis (TGA-51 Shimadzu, Tokyo, Japan) and DSC were used to assess thermal changes across a temperature range of 25–600 °C (Shimadzu DSC-60 Plus, Tokyo, Japan). Raman spectrometer (Shimadzu IR–Tracer 100 Fourier Transform Infrared Spectrophotometer, Tokyo, Japan) was used to determine the Raman spectra of the BT-An@Zetag composite (HOUND UNCHAINED LABS spectrometer, Berlin, Germany). A NOVA 4200e was used to evaluate the nitrogen adsorption–desorption isotherms at 77 K (Quantachrome Instruments, Boynton Beach, FL, USA). The Brunauer–Emmett–Teller (BET) equation and the specific surface area were used to further investigate surface area and pore size.Also, Pore size distributions were also calculated using the Barrett–Joyner–Halenda (BJH) methods. Initial and final concentrations of arsenate were analyzed using Triple Quadrupole Inductively Coupled Plasma Mass Spectrometry (TQ-ICP-MS), Thermo Fishers scientific (Waltham, MA, USA).

### 3.4. Batch Experiments

The batch system was used to optimize the adsorption process of As(V) onto BT-An@Zetag composite surfaces to determine the best adsorption conditions. The effects of adsorbent dose (5–55 mg/L), solution pH (2–9) adjusted with HCl and NaOH, agitation rate (50–250 rpm), and temperature (25–45 °C) at different residence times were investigated using different initial As concentrations (5–60) mg/L prepared by diluting a stock solution of As(V) of 1000 mg/L (5–150 min). A digital shaker (ELMI/Europe—Model: DOS-20L) was used to agitate similar weights of the composite (20 mg) with 50 mL of As(V) solution for 120 min at constant temperature (25 °C) in a typical equilibrium experiment. The adsorbate/adsorbent mixture was separated for 30 min at 10,000 rpm using a centrifuge (Hettich, EBA 200 S, Hettich, Germany). The residual As(V) content was measured using an ICP (Model DR6000, HACH/Germany). Triplicate experiments were used to determine the removal efficiency (R%) and adsorption capacity (q_e_, mg/g) following Equations (1) and (2), respectively [25,28]:

(1) $$R\%=\frac{C_{0}-C_{e}}{C_{0}}\times 100$$

(2) $$q_{e}=\frac{\left(C_{0}-C_{e}\right)V}{W}$$

where C_0_ and C_e_ are the As(V) initial and equilibrium concentrations (mg/L), respectively. R is the removal percentage, q_e_ is the adsorption capacity, V is the As(V) volume (L), and W is the adsorbent weight (g).

### 3.5. Kinetics, Isotherms, and Thermodynamics

To study the adsorption paths, mechanisms, and rates, four kinetic models were used for experimental data fitting (e.g., pseudo-first-order, pseudo-second-order, Elovich, and intraparticle-diffusion, Equations (3)–(6), respectively) [25,39]:

(3) $$\ln\left(q_{e}-q_{t}\right)=\ln q_{e}-k_{1}t$$

(4) $$\frac{t}{q_{t}}=\frac{1}{k_{2}q_{e}^{2}}+\frac{t}{q_{e}}$$

(5) $$q_{t}=\beta \ln\left(\alpha \beta \right)+\beta \ln t$$

(6) $$q_{t}=K_{p}t^{0.5}+C$$

where q_e_ and q_t_ (g/g) are the equilibrium and time adsorption capacities, respectively. The constant rate parameters are represented by k_1_ (min^−1^) and k_2_ (g/gmin) and are related to the pseudo-first-order and pseudo-second-order models, respectively. The number of possible active sites (g/g) is equal to the adsorption extent (g/g min). The rate constant (g/g min) and intercept are K_p_ (g/g min) and C, respectively. The adsorbed solute fraction of time t is F = q_t_/q_e_.

Isothermal models were used to examine the adsorption isotherm to demonstrate the interaction between As(V) and the fabricated BT-An@Zetag composite [40]. To illustrate the adsorption equilibrium data, the Langmuir, Freundlich, and Temkin isotherm models, Equations (7)–(9), were fitted and studied.

(7) $$\frac{C_{e}}{q_{e}}=\frac{1}{q_{\max}K_{L}}+\frac{C_{e}}{q_{\max}}$$

(8) $$\ln q_{e}=\ln K_{F}+\frac{1}{n}\ln C_{e}$$

(9) $$q_{e}=B_{T}\ln\mathrm{KT}+B_{T}{\mathrm{lnC}}_{e}$$

The q_max_ (g/g) and K_L_ (L/g) represent the saturated adsorption capacity and the Langmuir isotherm constant, respectively. The adsorption capacity is represented by K_F_, while the adsorption intensity is represented by 1/n. The heat of adsorption (J/mol) is represented by B, while the maximum binding energy (L/g) is represented by KT. The adsorption favorability could be described using the dimensionless separation factor (RL, see Equation (10) [28,41]:

(10) $$R_{L}=\frac{1}{1+K_{L}C_{0}}$$

The interior energy change that transpires in the adsorption of As(V) ion using the fabricated adsorbent, as well as spontaneity, and reaction paths were investigated using the thermodynamic parameters (e.g., Gibbs free energy (G), entropy (S), and enthalpy (H), expressed by Equations (11) and (12) [38].

(11) $$\ln K_{D}=-\frac{\Delta H}{\mathrm{RT}}+\frac{\Delta S}{R}$$

(12) ΔG=ΔH−TΔS

### 3.6. Reusability Test

The manufactured BT-An@Zetag composite was tested for reusability by soaking it in 1 M HNO_3_ for 6 h where the number of cycles in the adsorption–desorption process wascounted.

## 4. Conclusions

The propertyof the manufactured BT-An@Zetag composite for the adsorption of As(V) from the aqueous solution was demonstrated in this investigation. Various characterization technologies were used to thoroughly characterize the produced adsorbent. Solution pH, contact time, adsorbent dosage, agitation rate, initial metal ion concentration, and temperature were all evaluated and optimized as factors impacting As(V) absorption. The isotherm results revealed that As(V) is adsorbed on BT-An@Zetag as a monolayer where the maximum capacity of adsorption is38.8 mg/g at pH 3. The kinetics results also showed that the pseudo-second-order model adequately described the adsorption process. More than one mechanism was implicated in the adsorption of arsenic onto BT-An@Zetag, according to the Bedsides, Elovich, and intraparticle diffusion. The thermodynamic parameters such as ∆H and ∆G indicate that chemical, endothermic, and entry-driven adsorption is the most likely mechanism. Furthermore, the regeneration and reusability studies revealed that even after four cycles, the BT-An@Zetag adsorbent maintained good adsorption behavior. As a result, the BT-An@Zetag composite might be employed for the effective removal of As(V) from wastewater as well as being reusable.

## Figures and Tables

**Table 1:** Textural parameters of the BT-An@Zetag composite.

Composite	SBET (m^2^/g)	Total Pore Volume (Vt) (cm^3^/g)	Mean Pore Width (nm)
BT-An@Zetag	81.935	0.0333	13.87

**Table 2:** Kinetic model adsorption variables for the adsorption of As(V) on the BT-An@Zetag composite.

Kinetic Model	Parameter	Value
Pseudo-first-order	q_e,cal_(mg/g)	38.65
	q_e,exp_ (mg/g)	26.2
	K1 (min^−1^)	0.026
	R^2^	0.925
Pseudo-second-order	q_e,cal_(mg/g)	38.65
	q_e,exp_ (mg/g)	41.66
	R^2^	0.940
Elovich model	α (mg/g min)	0.135
	β (mg/g)	34.293
	R^2^	0.907

**Table 3:** Thermodynamic parameters of the adsorption process based on the Van’t Hoff plot.

Parameter	∆H (KJ/mole)	∆S (J/mol K)	∆G (KJ/mole)
	36.864	−107.53	67.934 to 71.058

**Table 4:** Isotherms’ model parameters for the As(V) adsorption on BT-An@Zetag composite.

Langmuir				Freundlich			Temkin		
q_max_ (mg/g)	K_L_ (L/mg)	R^2^	R_L_	k_f_ (mg/g)	1/n	R^2^	B_T_ (J/mol)	K_T_ (L/mg)	R^2^
38.65	0.606	0.978	0.367	2.535	0.7502	0.997	12.96	0.093	0.839

**Table 5:** Comparison of the maximum adsorption capacity of As(V) for adsorbents from selected studies with the BT-An@Zetag composite.

Adsorbent	q_max_ (mg/g)	Reference
Mesoporous alumina	36.6	[24]
NdFeO_3_	126.58	[59]
Granular activated alumina	15.9	[18]
Porous hematite	5.67	[36]
Iron-Based Adsorbent	7.0	[37]
Modified biochar	56.06	[60]
Magnesium ferrites	45.52	[61]
Fe–Mn modifed biochar	8.25	[62]
BT-An@Zetag	38.65	This study

## Data Availability

Not applicable.

## Sample Availability

Samples of the compounds are not available from the authors.
